# G Protein-Coupled Receptors at the Crossroad between Physiologic and Pathologic Angiogenesis: Old Paradigms and Emerging Concepts

**DOI:** 10.3390/ijms18122713

**Published:** 2017-12-14

**Authors:** Ernestina M. De Francesco, Federica Sotgia, Robert B. Clarke, Michael P. Lisanti, Marcello Maggiolini

**Affiliations:** 1 Department of Pharmacy, Health and Nutrition Sciences, University of Calabria via Savinio, 87036 Rende, Italy; ernestina.defrancesco@manchester.ac.uk; 2 Breast Cancer Now Research Unit, Division of Cancer Sciences, Manchester Cancer Research Centre, University of Manchester, Wilmslow Road, Manchester M20 4GJ, UK; robert.clarke@manchester.ac.uk; 3 Translational Medicine, School of Environment and Life Sciences, Biomedical Research Centre, University of Salford, Greater Manchester M5 4WT, UK; fsotgia@gmail.com (F.S.); michaelp.lisanti@gmail.com (M.P.L.)

**Keywords:** GPCR, tumor angiogenesis, tumor microenvironment, VEGF, HIF-1, GPER, SDF-1, sphingosine-1P

## Abstract

G protein-coupled receptors (GPCRs) have been implicated in transmitting signals across the extra- and intra-cellular compartments, thus allowing environmental stimuli to elicit critical biological responses. As GPCRs can be activated by an extensive range of factors including hormones, neurotransmitters, phospholipids and other stimuli, their involvement in a plethora of physiological functions is not surprising. Aberrant GPCR signaling has been regarded as a major contributor to diverse pathologic conditions, such as inflammatory, cardiovascular and neoplastic diseases. In this regard, solid tumors have been demonstrated to activate an angiogenic program that relies on GPCR action to support cancer growth and metastatic dissemination. Therefore, the manipulation of aberrant GPCR signaling could represent a promising target in anticancer therapy. Here, we highlight the GPCR-mediated angiogenic function focusing on the molecular mechanisms and transduction effectors driving the patho-physiological vasculogenesis. Specifically, we describe evidence for the role of heptahelic receptors and associated G proteins in promoting angiogenic responses in pathologic conditions, especially tumor angiogenesis and progression. Likewise, we discuss opportunities to manipulate aberrant GPCR-mediated angiogenic signaling for therapeutic benefit using innovative GPCR-targeted and patient-tailored pharmacological strategies.

## 1. Introduction

Over the past decade, the discovery and study of G-protein coupled receptors (GPCRs) has unveiled novel molecular mechanisms through which extracellular signals promote changes in cell functions. Encoded by more than 900 genes in the human genome, GPCRs belong to one of the largest families of membrane proteins involved in the detection of a wide panel of extracellular stimuli, including photons and ions, as well as peptides, proteins, hormones and phospholipids [1,2]. Members belonging to the GPCR superfamily regulate a broad spectrum of physiological functions, such as vision, smell, and taste, as well as neurological, cardiovascular, endocrine, and reproductive functions [1,2,3].

**Figure 1:**
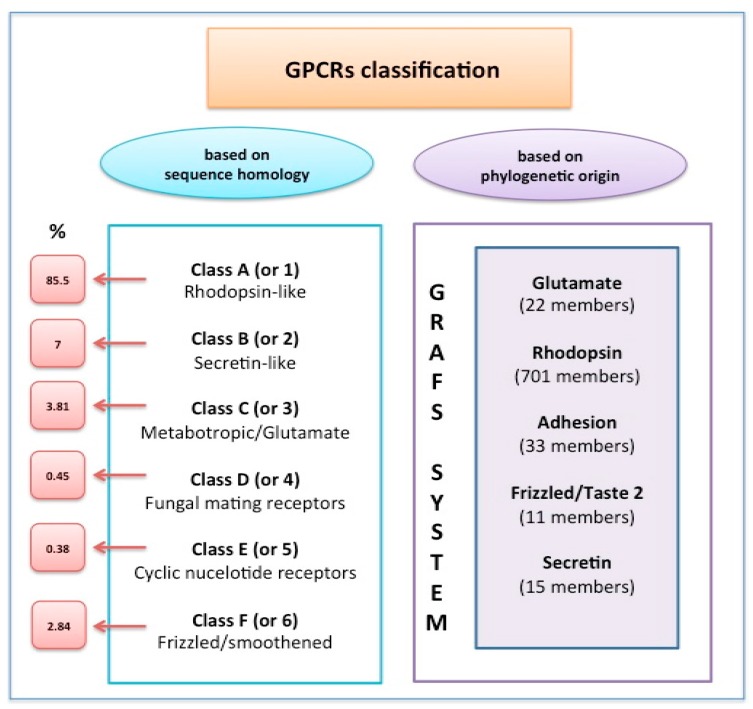
G-protein coupled receptors (GPCRs) classification. The International Union of Pharmacology (IUPHAR) classification (left column) applies to both vertebrates and invertebrates (Class D and E are unique to invertebrates). The GRAPH system (middle column) applies specifically to vertebrates.

GPCRs signal through their association with G-proteins, which are membrane bound heterotrimers consisting of a guanine diphosphate (GDP)-bound α subunit with GTPase activity, and a Gβγ functional monomer [1,2,3]. Based on the sequence of the α subunit, G-proteins are defined in four classes (Gαs, Gαq, Gαi, and Gα12), each coupling to more than one receptor subtypes, although with different affinity [2]. GPCRs are classified based on their phylogenetic origin and sequence homology (Figure 1).

Typically, upon ligand binding, the receptor undergoes a conformational change transmitted to the heterotrimer, whose tri-dimensional rearrangement determines the exchange of GTP for GDP on the α subunit and the dissociation of the Gβγ monomer. Overlapping with GTP recruitment, the transition from “off” to “on” state allows the Gα and Gβγ subunit to interact with and regulate the activity of a vast repertoire of transduction effectors, such as calcium, adenylyl cyclase, phospholipase C, phosphodiesterases, and protein kinases [4,5]. These second messengers activate additional intracellular pathways such as the mitogen-activated protein kinase (MAPK), phosphoinositide-3 kinase (PI3K)-Akt, small GTP-binding proteins (Ras and Rho GTPases), which ultimately engage transcription factors, leading to gene expression changes [5,6]. Additionally, many seven trans-membrane receptors (7TMRs) are orphan GPCRs with unidentified ligands and potential ligand-independent properties, suggesting that GPCR-mediated action may include unexpected biological functions, conceivably unrelated with the classical paradigm of a signal conveyed through the cell membrane [7]. In addition to their participation in an extraordinary number of physiological processes, some of which yet to be defined, GPCRs are implicated in the onset and evolution of several pathological conditions, including inflammatory and degenerative diseases, metabolic imbalances, and cancer [8,9,10,11]. Not surprisingly, the manipulation of aberrant GPCR transduction pathways holds great therapeutic potential [12]. Indeed, GPCR-targeting agents, totaling almost one third of all currently marketed drugs, are excellent candidates for the treatment of a widespread array of diseases [12,13,14].

For instance, GPCRs as drug targets to halt the anomalous formation of new blood vessels may be a strategy to normalize the dysregulated vascularization, which distinguishes various ischemic and inflammatory conditions and cancer [15]. Neoangiogenesis is an essential physiological and developmental process tightly regulated by a highly coordinated balance between pro- and anti-angiogenic factors. Alterations in this fine-tuned equilibrium could arguably lead to the increased production of pro-angiogenic mediators and the loss of inhibitory factors, which ultimately drive an aberrant vascularization [16,17,18]. For example, during cancer progression, an excessive vascular growth with abnormal vessel remodeling and tortuosity is actively stimulated by the release in the tumor microenvironment of angiogenic mediators such as vascular endothelial growth factor (VEGF) [19,20,21].

Several 7TMRs are reported to trigger VEGF release to boost the formation of new blood vessels, particularly during cancer evolution toward an aggressive and metastatic cancer phenotype [22,23], suggesting that GPCRs may serve as novel drug candidates in combination therapies aimed at combatting tumor angiogenesis. In addition to their role in the production of angiogenic factors, 7TMRs also function as receptor for angiogenic ligands, directly mediating endothelial cell function. Hence, GPCRs are regarded as potent regulators of blood vessel formation, in both developmental and pathological angiogenesis. Based on these observations, a better understanding of the molecular mechanisms orchestrated by GPCR signaling and driving the transition from a non-angiogenic to an angiogenic phenotype may pave the way for the control of a plethora of pathologic states governed by unbalanced angiogenesis, whose clinical management is not fully met.

Here, we review the angiogenic actions elicited by GPCRs and associated signaling partners in physio-pathological conditions such as ischemic and inflammatory disease (Section 2, summarized in Table 1 and Table 2). In particular, we comprehensively report the most recent findings on the GPCRs involved in vasculogenesis through the activation of developmental pathways, as well as on the most clinically relevant GPCRs involved in the regulation of angiogenesis both in normal and pathological conditions such as sphingosine 1P receptor (S1P1) and thrombin receptor (PAR1). Moreover, we analyze the most recent data on the role of GPCRs in regulating tumor angiogenesis, particularly focusing on the role of chemokine receptor CXCR4 and the G-protein estrogen receptor (GPER) (Section 3, summarized in Table 3). Finally, we discuss the opportunities to translate these major findings in a clinical setting. 

## 2. G Protein-Coupled Receptors (GPCRs) Involved in Early Vascular System Development and Maintenance: The Road to Cell Fate Commitment through Lineage Restriction

The vascular system fulfils the requirements of oxygen and nutrients of the whole organism and provides an efficient gateway for immune surveillance [17,18]. Thus, it is not surprising that structural or functional vessel abnormalities may contribute to the development of several pathological conditions. For instance, alterations in vessel maintenance play a causal role in the development of myocardial infarction, stroke, neurodegenerative and metabolic disorders, whereas an excessive vascular growth and permeability characterizes inflammatory conditions and malignant disease [17,18]. Hence, the identification of the multiple mechanisms orchestrating the complex process of blood vessel formation is relevant to the management of several ailments.

Vasculogenesis is usually referred to as the formation of primitive blood vessels during the early embryonic development [18]. It is a multifaceted process involving the in situ assembly of mesoderm-derived endothelial precursors (angioblasts) into a primitive vascular network [73]. In this context, several developmental signals have been considered as the main drivers of mesoderm progressive restriction to the endothelial lineage, nonetheless ECs may also derive from hemangioblasts, bipotential precursors that give rise to both hematopoietic and endothelial cells [74]. These initial steps are followed by vessel sprouting, characterized by the recruitment of pericytes and vascular smooth muscle cells that enwrap the primitive tubule of endothelial structures, giving rise to arteries and veins [75,76]. This latter process of vessel splitting from pre-existing vessels is known as angiogenesis, which actively participate to vascular maintenance both pre- and postnatally. Despite the elevated angiogenic activity of the early developmental phases, in the adult, blood vessels sprout new branches very rarely, mostly remaining in a quiescent state. Of note, certain angiogenic signals may waken up and activate endothelial cells, toward a motile and invasive phenotype, with increased protrudent filopodia [18]. In these conditions, tip cells and stalk cells start to organize new sprouts, establish a lumen and build vessel loops. The process culminates with the formation of basement membranes and the recruitment of mural cells, which stabilize the novel vessels into efficiently working structures [77,78]. When the need for new blood vessel is met, proangiogenic signals are ablated and quiescence is restored [77,78].

GPCRs participate in all the aforementioned steps involved in vasculogenesis and angiogenesis; indeed, their expression has been detected in endothelial, vascular smooth, tip cells, as well as in endothelial progenitors [79]. For instance, Wnt signaling has become increasingly recognized for its role in developmental processes such as vascular differentiation, and has been shown to be necessary for the differentiation of hepatic sinousoidal endothelial cells (HSECs) [24]. In addition, the ligands Wnt7a and Wnt7b, derived from neural progenitors, have been demonstrated to activate the Wnt/β-catenin signaling in ECs, leading to angiogenesis and blood–brain barrier (BBB) formation [25,26,80]. Wnt-dependent angiogenic responses have also been observed in human foetal aorta-derived CD133^+^ cells and correlated with tissue repair in a pre-clinical model of ischemic diabetic ulcer [27]. Recently, a method for the production of human progenitor stem cells (HPSC)-derived endothelial progenitors and ECs based on small-molecule activation of Wnt signaling has been described, corroborating the involvement of this pathway in lineage restriction toward ECs, and suggesting that the manipulation of Wnt/β-catenin signaling may serve as an effective in vitro method for the generation of ECs to study vascular development and disease [28].

The Wnt receptors, Frizzled-4 and Frizzled-7, have been implicated in EC differentiation and complete vascularization of the neural retina [81,82], while in human placenta, Frizzled-9 enriches for mesenchymal stem cells (MSCs) with arteriogenic and angiogenic properties [83]. Evidence for the importance of sonic hedgehog (Shh) pathway in ECs formation has been reported several times. Indeed, this developmental pathway has been correlated with endothelial progenitors activation, arterial gene expression, vessel sprouting, and adult hematopoietic stem cell (HSC) formation [84,85]. All these responses appear to be affected by age, as down-regulation of desert hedgehog (hh) and smoothened (Smo) impairs hh signaling in aged mice, ultimately leading to inhibition of angiogenesis, as demonstrated also using a Smo haplo-insufficiency mice model [86]. Interestingly, the integrated connections between hh and other developmental and angiogenic cascades such as Notch and VEGF has been shown to instruct ECs toward an arterial or a venous differentiation, whereas the interaction between hh, Notch and Scl drives a lineage shift characterized by hematopoietic progenitor formation and endothelial-to-hematopoietic transition [87,88]. Supporting these observations, in animal models of brain ischemic injury and stroke, the pharmacological activation of the hh pathway has proven therapeutic benefit by promoting tissue repair and angiogenic programs, associated with increased functional recovery [89,90].

GPCRs other than the ones classically involved in self-renewal, quiescence and differentiation have been implicated in the endothelial differentiation and in the hematopoietic process, which together contribute to maintain homeostasis within the vascular system. For instance, a GPCR gene expression screen performed in mouse embryonic stem cells (mESCs) has identified PAR1 as the crucial GPCR involved in cell reprograming toward an endothelial phenotype [29]. Extending these observations, regulation of lineage specification and homing, together with preservation of self-renewal capacity and expansion of hematopoietic stem and progenitor cells (HSPCs) are regulated by several members of the rhodopsin family, such as chemokine C-X-C motif receptor 4 (CXCR4), sphingosine-1-phosphate receptor (S1PR1/EDG1), and prostaglandin receptor (EP2), and a member of the glutamate family (calcium sensing receptor CaSR) [91,92,93,94,95,96]. Together, these data highlight the involvement of GPCRs in the activation of the hematopoietic process, in the formation and maintenance of a proper vascular bed and in cell reprograming toward endothelial lineage.

These observations suggest that the identification and manipulation of GPCR-mediated developmental pathways may be an effective strategy in regulating the early stages of angiogenesis. Based on this hypothesis, Kaur and co-workers [97] recently reported that GPCR expression is heterogeneous but functionally patterned within the vascular system, with specific GPCR expression clusters observed in pathological conditions [97]. More specifically, by using a microfluidic-based single-cell GPCR expression analysis in both primary smooth muscle cells (SMCs) and endothelial cells (ECs), the authors demonstrated that although the GPCR expression is heterogeneous in normal conditions, several subpopulations expressing specific GPCRs patterns can be detected upon stressful stimuli, such as inflammation, sepsis or atherosclerosis [97]. Furthermore, several GPCRs such as Gpr39, Gprc5b, Gprc5c or Gpr124 were up-regulated during SMC dedifferentiation, suggesting that the changes in GPCR repertoire might represent a crucial event in the transition from healthy to altered vascular cells [97]. These observations clearly indicate that GPCRs other than the ones involved in the regulation of developmental pathways may play a pivotal role not only in vasculogenesis, but also in triggering new blood vessel formation in pathological conditions. In Section 2.1 and Section 2.2, we describe Shingosine 1P Receptors and Thrombin Receptors as examples of well-acknowledged GPCRs involved in the regulation of physio-pathological angiogenesis.

### 2.1. Lysophospholipid Receptors: Angiogenic Actions Mediated by SPHINGOSINE 1P Receptors

Sphingosine-1-phosphate (S1P) belongs to the class of lysophospholipids with sphingoid backbone, deriving from the metabolism of sphingomyelin by the spyngomielinase pathway [98,99]. S1P, which is generated by the ATP-dependent phosphorylation of sphingosine triggered by the enzyme sphingosine kinase, is involved in the transmission of cell signals that regulate critical biological responses, such as proliferation, cell motility, and apoptosis [100,101,102,103]. Although functioning as an intracellular mediator, S1P can be released and exert its effects extracellularly, binding to cell surface receptors [104]. Indeed, S1P is a bona-fide ligand for the G-protein coupled receptor endothelial differentiation gene (*Edg*) family members, also known as S1P receptors [104,105]. The Edg family comprises five receptors, S1P1 (*Edg-1*), S1P2 (*Edg-5*), S1P3 (*Edg-3*), S1P4 (*Edg-6*) and S1P5 (*Edg-8*), which can couple to different G proteins and activate several downstream signaling pathways, including the ERK, JNK, small GTPases such as Rho and Rac, phospholipase C, adenylyl cyclase and phosphatidilinositol 3 kinase (PI3K) [104,105]. S1P1 is considered an important mediator for vascular maturation during embryonic development, as demonstrated by gene ablation in vivo [105,106]. In this respect, several vascular defects incompatible with life were detected in the early stage of embryonic development in S1P knock-out mice, characterized by an altered migratory capability of mural cells (VSMCs) and pericytes around the nascent vessels [37]. In vitro and in vivo studies have provided evidence on the ability of S1P/S1P1 signaling to activate an angiogenic program in ECs, by stimulating cell proliferation, migration, tube assembly and formation of cell junctions [107,108]. In addition to its remarkable ability in preserving endothelial function, S1P acts as a mitogen and migratory factor on VSMCs [38,39], corroborating that S1P and its receptors play an integral role in vessel maturation, development and repair. S1P/S1P1 signaling exerts a cardiovascular beneficial action in diverse ischemic conditions, both during pre- and post-conditioning, although the protective effects have been mainly attributed to the activation of salvage kinase pathways, the regulation of mitochondrial dynamics and the inhibition of the RAS (renin-angiotensin system) signaling [41,42,43,109]. Nonetheless, several reports have demonstrated that both endogenous and microparticles-delivered S1P exerts beneficial actions linked to angiogenesis and blood stream recovery in animal models of limb ischemia [110,111].

In addition, impairment of S1P-dependent angiogenic actions has been detected during preeclampsia, a serious pregnancy disorder associated with maternal and foetal morbidity and mortality [112]. Based on these observations, the administration of S1P1 agonists such as FTY720 and LASW1238 has been proven effective in mice and proposed as novel therapeutic strategy in the clinical management of ischemic injury, although in humans the clinical use of FTY720 is complicated by severe adverse side effects [43,109,110,111,112,113].

On the other hand, S1P has been reported to mediate detrimental actions attributable to the release of pro-inflammatory mediators such as IL-17A in cerebral ischemia [114,115]. Likewise, the activation of an S1P2-dependent inflammatory program has been regarded as one of the leading route of pathologic neovascularization in mice retina [44]. Together, these findings suggest that S1P signaling may differentially promote beneficial effects related to angiogenesis in pathological conditions, depending on various factors such as tissue specificity and the GPCR subtype involved.

### 2.2. Thrombin Receptors

Thrombin is a serine protease that plays a key role in platelet aggregation and clot formation by converting fibrinogen in fibrin, thus allowing tissue repair and wound healing [116]. In addition to hemostasis, thrombin orchestrates several physio-pathological responses in the vascular system such as angiogenesis and atherosclerosis, by stimulating platelets, ECs, and VSMCs [116,117]. These cellular, non-hemostatic actions of thrombin are primarily mediated by its interaction with a family of GPCRs named Protease-Activated Receptors (PARs), which are activated by proteolytic cleavage and subsequent unmasking of a new tethered N-terminal residue [30,117]. This signaling mechanism, unique within the GPCR superfamily, is shared among all four members of thrombin receptors (PAR1-PAR4), which show differential agonist specificity and signaling properties. In fact, only human PAR1, PAR3 and PAR4 can be activated by thrombin, while PAR2 is cleaved mainly by trypsin and other coagulation factors [31]. The activation of PARs results in the engagement of a wide-ranging network of signaling pathways, including G12/13, Gq and Gi/z proteins as well as small G-proteins such as Rho. These signaling network are mainly involved in cytoskeletal remodeling in platelets as well as migration and permeability of ECs [30,31,32]. Through the involvement of Gαq, PARs can trigger phospholipase Cβ-dependent calcium mobilization and protein kinase C activation, involved in granule secretion, platelet aggregation and transcriptional responses in ECs, while Gαi signaling and Gβγ subunit are mainly responsible for the regulation of adenilate cyclase and PI3K activity [32,118,119].

Beyond the regulation of vessel tone and permeability, PARs signaling in ECs plays a pivotal role in the early stages of vasculogenesis. Indeed, ablating PAR-1 expression in ECs was sufficient to prevent a proper vascular development and maturation in mouse embryos, while rescuing PAR-1 expression reversed the vascular abnormalities [33]. Recently, Huang et al. demonstrated that PAR1 activation stimulates platelets to selectively release diverse pro-angiogenic regulators, including VEGF, stromal cell-derived factor 1α, and matrix metalloproteinases, which in turn trigger the angiogenic activity of endothelial progenitor cells (EPCs) [34]. Likewise, in ECs thrombin has been shown to activate HIF-1α/VEGF signaling, to increase the expression of VEGF receptors (VEGFRs), to promote the secretion of angiopoietin-2 and to trigger cell proliferation and tube formation [35,36,120]. Next, using the chick chorioallantoic model as a physiologically intact in vivo system, thrombin has been shown to engage PAR1/MAPK/AKT transduction pathway to promote neoangiogenesis [121]. Interestingly, the angiogenic effects of thrombin have been explored for their beneficial cardiovascular effects, particularly in low oxygen conditions. For instance, systemic administration of the thrombin peptide TP508 during chronic hypoxia and ischemia has been shown to potentiate the angiogenic response of aortic endothelial cells to VEGF [45]. Corroborating these findings, thrombin triggered angiogenesis and cardiac recovery in a model of acute myocardial infarction [46]. Based on these findings, patients affected by ischemic conditions might get benefit from the administration of thrombin or its derivatives, which would stabilize the blood vessel network through PAR1 stimulation. On the other hand, aberrant signaling through the thrombin/MMP1/PAR1 transduction pathway and its correlation with VEGF expression has been detected in patients affected by proliferative diabetic retinopathy [47]. In addition, in human retinal microvascular endothelial cells the stimulatory effects induced by thrombin and MMP1 through VEGF were prevented using the PAR1 inhibitor vorapaxar [47]. These findings, obtained using pre-clinical and animal models, point at the involvement of thrombin/PAR1 signaling in the pathogenesis of retinal disease, particularly upon certain metabolic unbalances such as diabetes, suggesting that targeting PAR1 may represent a novel and safe tool toward the eradication proliferative diabetic retinopathy (PDR). In summary, these findings suggest that the manipulation of PAR signaling holds promise for therapeutic benefit in certain inflammatory vascular conditions characterized by ischemia, hypoxia, as well as hyperglycemia.

## 3. GPCR Signaling and Tumor Angiogenesis: An Interactive Loop Promoting Disease Progression

**Figure 2:**
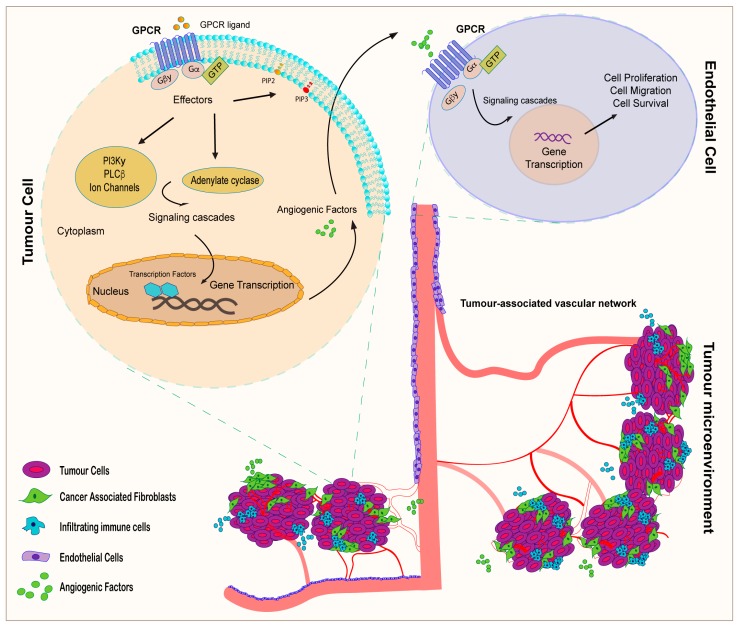
GPCRs involved in tumor angiogenesis. GPCR signaling mediates the cross-communication between stromal, endothelial and cancer cells toward neoangiogenesis and tumor progression.

Establishment of an amplified blood vessel network is an essential prerequisite for cancer growth and progression, as tumor neo-vascularization provides cancer cells with adequate oxygen and nutrient supply to satisfy the enhanced metabolic requirements of the neoplastic mass [122]. Multiple signaling mediators contribute to the regulation of tumor angiogenesis, an intricate process that entails the functional cooperation of cancer cells and several components of the microenvironment [123]. First, ECs are activated toward a secretory phenotype characterized by the release of proteases, which promote the degradation of the basement membrane, hence generating an interstitial space for the migration and proliferation of ECs. Such biological responses precede the endothelial tube assembly, which represents the main step toward the formation of new blood vessels [122,123]. Unlike normal blood vessels, tumor vasculature is characterized by a disorganized labyrinth of maladapted and abundant vessels [122]. The impairment in vascular structure and function is evidenced by the establishment of tortuous blood vessels, increased leakiness, permeability and extravasation. Altogether, these vascular abnormalities contribute to intra-tumor hypoxia, which is considered a distinguishing feature of locally advanced solid cancer. Low oxygen tension usually results from an imbalance between oxygen (O_2_) supply and consumption [124]. Multiple biochemical and mechanical forces within the microenvironment contribute to intra-tumor hypoxia, which is observed in cells distant more than 70–150 μM from a perfused blood vessel. The rapid expansion of the tumor mass is mainly responsible for the increased diffusion distance between nutritive blood vessels and the cells lying in the central regions of the neoplastic formation. Furthermore, a reduced O_2_ transport capacity is due to the loss of red blood cells, caused by extravasation and aggravated by several anti-cancer treatments [124]. To escape the hypoxic hostile microenvironment, cancer cells activate a plethora of biological responses such as angiogenesis, thus establishing a vicious circle in which tumor hypoxia triggers aberrant blood vessel formation toward the development of malignant disease progression [124]. In accordance with this scheme, it has been demonstrated that hypoxia, altering gene expression and/or regulating post-transcriptional and post-translational events, generates relevant changes in the tumor cell proteome in order to overcome the low oxygen stress [124,125]. To a large extent, quantitative proteomic analysis has demonstrated that proteins involved in the activation of angiogenic programs are actively produced in tumor cells exposed to hypoxia [126]. The vascular endothelial growth factor (VEGF) is the most relevant pro-angiogenic factors in both physiological and pathological angiogenesis [126]. In cancer cells, the regulation of VEGF expression is stimulated by the hypoxia inducible factor-1 (HIF-1), a transcription factor that mediates cell adaptation to low oxygen conditions [127,128]. Indeed, overexpression of HIF-1α/VEGF signaling is associated with increased vascular density, higher tumor grade, therapeutic resistance and poor prognosis [126,129]. Due to its role in supporting angiogenesis, the HIF-1α/VEGF signaling pathway is currently regarded as an outstanding pharmacological target to control aberrant vessel sprouting in cancer [130]. Diverse members of the GPCR superfamily have been shown to mediate angiogenic signals in cancer, suggesting that the manipulation of GPCR signaling may be included among the novel therapeutic strategies to halt angiogenesis in the tumor microenvironment [131,132,133]. Several GPCR ligands and accessory proteins have been shown to contribute to tumor vascularization, including lysophosphatidic acid (LPA), sphingosine 1P, thrombin, angiotensin, prostaglandin, melatonin and diverse interleukin and chemokines [22,23], suggesting that GPCR-mediated signaling may support vessel formation in diverse tumor types (Figure 2). For instance, neuroblastoma cells engineered to express a constitutively active mutant of LPA receptor 1 (LPAR1) showed an increased VEGF expression and ability to promote ECs migration respect to the normal counterpart [134]. Additionally, both LPAR1 and LPAR3 have been implicated in multiple myeloma vascularization [134], while LPAR1 has been shown to boost angiogenic response in pre-neoplastic lesions, thus suggesting that the involvement of these receptors in the transition from dysplastic to neoplastic formations is correlated with increased angiogenesis [135]. Supporting these findings, Song and collaborators [51] found that, in ovarian cancer cells, LPA stimulates the HIF-1 independent expression of VEGF, through a G12/13-Rho-Rock-c-myc mediated mechanism [51]. Likewise, a pan-antagonist for 4 LPA, namely BrP-LPA, exhibited anti-growth and anti-angiogenic actions in a lung cancer xenograft model [136].

Several mediators involved in vessel repair and vascular tone maintenance have been shown to exert stimulatory actions in tumor development and progression, including Angiotensin II, which signal through the Angiotensin II type 1 receptor (AGTR1) to trigger angiogenic actions [137]. By using multiple independent breast cancer profiling studies, a MetaCOPA bioinformatics analysis has allowed prioritizing AGTR1 as a second ranked meta-outlier, immediately after HER2neu, which was identified as the most significant meta-outlier [137]. Indeed, overexpression of AGTR1 in breast cancer cells has been shown to promote tumor angiogenesis and epithelial mesenchymal transition (EMT), which together contribute to disease progression toward a malignant phenotype [52]. Extending these observations to additional tumor types, a reduction in capillary density was observed in AGTR1 deficient mice engrafted with melanoma cells [53]. Furthermore, the ANGII/AGTR1 transduction pathway was involved in the release of VEGF by tumor-associated macrophages, hence suggesting that host AGTR1 signaling triggers new blood vessel formation by acting on tumor microenvironment components [53]. While AGTR2 has been classically involved in antagonizing AGTR1-mediated actions, a recent study has demonstrated that the dual targeting of AGTR1 and AGTR2 using losartan and CGP42112A synergistically decreases cell survival and angiogenesis in epithelial ovarian cancer, through the inhibition of PLC β3 phosphorylation and VEGF expression [138].

Compelling experimental evidence obtained in vitro as well as animal models has revealed the role of sphingosine-1-phosphate (S1P) and its receptors (S1P1–S1P5) in generating a microenvironment permissive to cancer growth and progression [139]. Likewise, tumor vasculature highly expresses the S1P receptor type 1 (S1P1), and its ablation suppressed tumor angiogenesis and growth in vivo [139]. Accordingly, using as experimental system a mouse model of breast cancer, it was shown that deletion of S1P1 in tumor-associated macrophages reduces angiogenesis and lung metastatic spread, by inhibiting IL-1β signaling and inflammasome activation [54]. These observations suggest that the angiogenic actions elicited by S1P/S1P1 signaling may involve the active contribution of tumor microenvironment through the engagement of inflammatory cells and mediators. Spiegel and collaborators [140,141,142,143] have uncovered the molecular underpinnings of S1P action in several physio-pathological conditions, including cancer, thus helping to decipher S1P interaction with estrogen signaling toward breast tumor angiogenesis and progression [140,141,142,143]. As an intriguing outcome of these investigations, the S1P1 antagonist FTY720 has been shown to elicit strong anti-angiogenic effects and to inhibit breast cancer growth both in vitro and in vivo [144,145,146]. Furthermore, a combination therapy based on the oral administration of both sunitinib and FTY720 has proven to be effective in reducing tumor growth and normalizing blood vessels in a syngeneic breast cancer model [147].

In addition, virally encoded GPCRs such as the Kaposi’s sarcoma-associated herpes virus G protein-coupled receptor (KSHV-GPCR) exhibited angiogenic potential for its ability to induce the HIF-α/VEGF axis as well as EC survival [55,56]. These reports emphasize the importance of virally encoded GPCRs in promoting the survival of viral-infected cells and highlight the role of these receptors in the development of certain viral-related neoplastic disease [55,56].

### 3.1. Chemokine Receptors CXCR4 and CXCR5: A Signaling Hub in Tumor Angiogenesis

In humans, the family of chemokines includes 50 low molecular proteins with chemoattractant function and cytokine nature and 20 trans-membrane receptors coupled to G-proteins [148]. Primarily involved in leukocyte trafficking in homeostatic and pathologic conditions [148], chemokines and their receptors may also contribute to oncogenesis, being implicated in most of the processes characterizing the hallmarks of cancer [149]. Of note, chemokine signaling may serve as an efficient communication system between cancer cells and components of the tumor microenvironment such as stromal, immune and vascular cells. Indeed, chemokines and their receptors contribute to the multistep processes required for the angiogenic switch, starting from the early stages of progenitors mobilization and recruitment from bone marrow by SDF-1 (Stromal Derived Factor-1, also known as CXCL12), to the transdifferentiation of CSCs to endothelial phenotypes by CCL20 [150,151,152]. Some chemokines promote new blood vessel formation acting directly on ECs, as the CCR1-binding chemokines CCL15, CCL16 and CCL23 [153,154,155]. Nevertheless, many chemokines trigger angiogenesis by integrating paracrine signals within the tumor milieu [156]. For instance, CXCL17, CCL2 and CCL3 attract inflammatory cells such as monocytes, neutrophils and macrophages, and then induce angiogenic factors such as HIF-1α, VEGF and MMP-9 in the attracted cells [157,158,159,160]. As elevated levels of certain chemokines are associated with the formation of anomalous blood vessel network, anticancer strategies targeting chemokine signaling hold promise for the management of several malignancies characterized by aberrant vascularization. In parallel, the characterization of the molecular mechanisms involved in chemokine-dependent tumor angiogenesis is a topic under intense investigation, for its potential to unveil novel pharmacological target in cancer. In this context, using osteosarcoma cells as experimental model CCL3 was found to activate JNK, ERK and p38 and to trigger the down-regulation of miR-374b, which in turn promoted the up-regulation of VEGF and the VEGF-dependent migration and tube formation of EPCs [161]. Diverse microRNAs are involved in the up-regulation of VEGF levels mediated by CCR5. First, by analyzing gene expression from chondrosarcoma patients, a positive association was found between CCL5 and VEGF mRNA levels, whereas a negative correlation was assessed between CCL5 and miRNA200b expression [162]. Next, the stimulation of chondrosarcoma cells with CCL5 determined a PI3K-dependent down-regulation of miR-200b that resulted in increased VEGF protein levels [162]. It has also been reported that the angiogenic actions elicited by CCL5 in chondrosarcoma cells may rely on the down-regulation of miR-199a, which targets JAG-1 to up-regulate VEGF signaling thereby inducing EC differentiation [63,64]. Altogether these studies suggest that CCL5/CCR5 signaling regulates angiogenic responses in cancer by modulating certain miRNAs, nevertheless the stimulatory actions mediated by CCR5 may also rely on the direct regulation of the HIF-1α/VEGF transduction pathway [163]. Recently, it has also been established that the suppression of cancer cell-produced CCL5 or host CCR5 could result in defective breast tumor vascularization and growth [65]. For instance, the impairment in CCL5/CCR5 signaling abrogated angiogenesis by targeting the interactions between tumor and ECs, rather than halting the bone marrow derived activation of EPCs. These observations indicate that the abrogation of CCR5-mediated action may block tumor vascularization by hijacking microenvironmental communications, rather than altering EPC biology [65].

CXCL12 is a highly conserved chemokine that binds to CXCR4 as well as CXCR7 [164,165]. Experimental and clinical evidence have indicated that the CXCL12/CXCR4 axis promotes new blood vessel formation in diverse types of malignancies, including breast, ovarian, prostate, hepatic, gastric, colorectal cancer, and glioma [57,58,59,60,61,62,166]. Additionally, circulating CXCL12 levels have been associated with tumor angiogenesis in patients affected by multiple myeloma [167]. The angiogenic actions mediated by the CXCL12/CXCR4 transduction pathway in cancer may involve the activation of EPCs, as well as the recruitment of additional pro-angiogenic molecules such as VEGF. For instance, conditioned medium collected from osteosarcoma cells has been shown to promote the CXCL12-mediated migration of CD34+ progenitors [168]. Moreover, in gliomas characterized by high vasculogenic potential, the incorporation of EPCs into blood vessels was associated with CXCL12 secretion, whereas the CXCR4 antagonist AMD3100 abrogated the ability of CXCL12 to trigger EPC incorporation into the tumor vasculature [169]. On the other hand, CXCR4 activation triggered VEGF-dependent neoangiogenesis in glioma and breast cancer cells, suggesting that CXCL12 may synergize with other pro-angiogenic factors to induce the formation of new blood vessels [170,171]. Likewise, CXCR4 expression was detected up-regulated upon hypoxia, which is one of the main microenvironmental conditions involved in the activation of a compensatory angiogenic response in cancer [172].

As most chemokines, CXCL12 and its receptor may serve as connecting bridges between cancer and stromal cells, thus creating a permissive microenvironment for tumor growth and invasion. In this regard, cancer associated fibroblasts (CAFs) co-implanted with breast cancer cells stimulated tumor growth more efficiently than normal fibroblasts [173]. This effect has been at least in part attributed to the ability of CAFs to trigger tumor angiogenesis through the recruitment of EPCs mediated by CXCL12 [173]. The interaction between CXCL12 secreted by CAFs and CXCR4 expressed in cancer cells is also accountable for the direct growth effects elicited by activated stromal cells [173].

On the other hand, compelling experimental evidence suggest that CXCL12 from both epithelial and stromal cells may play a crucial role in breast tumorigenesis. In this vein, Liu and coworkers found drastic differences in the vascular content of mammary tumors induced by overexpression of Wnt1 compared with mammary tumors induced by overexpression of Her2 [174]. The increased tumor vascularization observed in MMTV-Wnt1 tumors and revealed by histological analysis was shown to be correlated with higher expression levels of CXCL12, both in tumor myoepithelial and stromal cells. In the same study, the administration of a CXCL12 neutralizing antibody was sufficient to inhibit the growth of Wnt1- but not Her2-overexpressing tumors and this effect was correlated with decreased infiltration of myeloid progenitor cells and ECs [174]. Based on these observations, anti-CXCL12 strategies may represent an innovative approach for targeting mammary tumors abundantly expressing myoepithelial and stromal CXCL12 [174].

The role of CXCR4 signaling in regulating key aspects of tumor progression such as angiogenesis has prompted the development of CXCR4 antagonists such as ADM3100, the first identified potent and selective CXCR4 antagonist [175,176]. ADM3100 prevents endothelial sprouting from human embryonic stem cells and efficiently antagonizes CXCL12-dependent angiogenic actions [177]. However, the administration of this drug in healthy volunteers has been shown to strongly increase the number of circulating EPCs and circulating angiogenic cells (CACs) deriving from monocytes [178], thus limiting the clinical use of ADM3100. Novel ligand-based approaches have led to the identification of alternative CXCR4 antagonists, such as peptide R, Nef-M1 and CTCE-9908 which elicited antiangiogenic activity in glioblastoma, breast, colon and prostate cancer [179,180,181].

### 3.2. Orphan GPCRs and Tumor Angiogenesis: The Case of GPER

To date, only 20% of the genes identified by the Human Genome Project as codifying for GPCRs have been matched with an endogenous ligand [182]. Indeed, deorphanization represents a challenging step toward the characterization of the physio-pathological functions of orphan 7TMRs [182]. Several approaches have been undertaken to unravel the biological actions of orphan GPCRs, including the phenotypical characterization after knocking-down or overexpressing the orphan protein, as well as the development of synthetic surrogate ligands [183]. However, many ligand-independent functions have been attributed to several orphan GPCRs, ranging from constitutive activity to hetero-dimerization with other proteins [183]. Based on these observations, the term “true orphan” refers to orphans with unknown ligands so far, while the term “conditional orphans” identifies GPCRs that can behave as orphans/non-orphans depending on the presence/absence of the ligand [183].

Several GPCRs involved in the regulation of angiogenesis do not possess an annotated ligand, nonetheless the contribution of these 7TMRs to vascularization is under the magnifying lens for the potential therapeutic implications. For instance, the orphan receptor GPR126 has been shown to participate in developmental and pathological angiogenesis by modulating VEGFR2 signaling [40]. In particular, among 100 orphan GPCRs screened, GPR126 expression was highly enriched in VEGFR2-positive cells isolated from a mouse embryoid body, which represents a useful animal model to study ESCs differentiation [40]. In the same study, GPR126 was found to regulate EC proliferation, migration and tube formation, as well as to contribute to vascularization in response to hypoxic stress [40]. The orphan GPR124 has also been implicated in new blood vessel formation, particularly in the tumor microenvironment. For instance, GPR124 contributed to VEGF-induced tumor vascularization by regulating cell-cell interaction, endothelial tube formation and permeability, cell migration and invasion [66]. In vivo, GPR124 knock-down in ECs ablated angiogenesis and growth in a mouse xenograft tumor model [66].

In an effort to discover a common angiogenic gene signature in human cancer, Masiero and co-workers analyzed more than 1000 primary human tumor samples and identified those genes whose expression jointly correlates with several well-known angiogenic genes in multiple cancer [67]. Furthermore, the authors complemented their analysis by studying the modulation of the gene signature in response to antiangiogenic treatments in preclinical models to identify novel potential uncharacterized players [67]. This approach allowed them to determine that the endothelial orphan 7TMR named ELDT-1 (Epidermal growth factor, latrophilin and seven-transmembrane domain-containing 1) is significantly up-regulated across multiple cancer types and correlates with tumor angiogenesis. In fact, ablation of ELDT-1 expression suppressed endothelial sprouting and vessel formation, and halted ovarian, colorectal cancer and glioblastoma growth [67,68,69].

The last orphan GPCR with angiogenic properties described in this review is the G-protein estrogen receptor (GPER), a member of the rhodopsin group of GPCRs [184,185]. GPER, also known as GPR30, was first cloned nearly twenty years ago and subsequently characterized for its ability to mediate estrogen action in several physio-pathological conditions [184,185,186]. GPER has been shown to regulate relevant biological functions in the reproductive, skeletal and central nervous system and to transduce signals involved in maintaining certain metabolic and immune responses [187,188,189,190,191].

Of note, GPER exerted cardiotropic actions and was implicated in the regulation of vascular biology and tone [48,49,192]. For instance, GPER expression was found increased in animal models of spontaneous and secondary hypertension, whereas the stimulation of GPER triggered beneficial cardiovascular effects and lowered blood pressure [50,193,194]. The protective effects triggered by GPER activation both in physiologic and pathologic conditions may mimic the beneficial actions of estrogens both in the heart and in the vascular network [195,196], suggesting that the manipulation of GPER transduction pathway could be considered in the clinical management of certain cardiovascular disorders [197,198].

Indeed, among the multifaceted aspects involved in GPER signaling, the contribution of this receptor to the mitogen actions elicited by estrogens in the development and progression of estrogen-sensitive tumors is notable, as well as contributing to pharmacological resistance to tamoxifen treatment [199]. As it concerns the molecular mechanisms of action, estrogenic GPER signaling engages several rapid transduction cascades such as MAPKs and PI3K/AKT, which in turn activate the intracellular transcriptional machinery, leading to stimulatory effects in cancer cells, as well as in components of the tumor microenvironment such as Cancer Associated Fibroblasts (CAFs) [200,201].

Intriguingly, based on our studies, GPER was included among the hypoxia-regulated genes within the breast tumor microenvironment [23,202]. In this regard, we provided evidence that a ROS-triggered and HIF-1α-dependent up-regulation of GPER may contribute to the adaptive breast cancer cell responses to low oxygen stress [23,202]. As hypoxia is mainly tackled by the generation of new blood vessels, we have explored the possibility that GPER may serve as a mediator of tumor angiogenesis. Hence, we have shown that a functional cooperation between HIF-1α and GPER drives the expression of VEGF in response to hypoxia and the heavy metal copper, which triggers biological actions as those observed in low oxygen conditions [70,71]. Furthermore, the engagement of the HIF-1α/VEGF axis by estrogen- and endothelin-activated GPER signaling has been shown to stimulate angiogenesis and tumor growth both in vitro and in vivo, as evidenced in breast and hepatic cancer models as well as in CAFs [72,203]. The above findings have been supported by other studies that correlate the expression of GPER with VEGF production and angiogenesis in neoplastic and inflammatory conditions [204,205]. In addition, GPER signaling promoted VEGF-independent blood vessel formation through diverse mechanisms, including the activation of the glycolytic enzyme phosphofructokinase-2/fructose-2,6-bisphosphatase 3 (PFKFB3), the up-regulation of acid ceramidase expression, the increase of X-linked inhibitor of apoptosis protein (XIAP), and the regulation of Na^+^/H^+^ exchanger-1 (NHE-1) activity [206,207,208,209]. In summary, these findings suggest that targeting GPER signaling in the interactions between cancer, stromal and endothelial cells could represent a novel anti-angiogenic strategy to halt aberrant vascularization in tumor patients.

## 4. Concluding Remarks and Challenges Ahead

Totaling over 900 members, GPCRs represent the largest and most targeted receptor family in the human proteome, with 25–30% of all drugs currently on the market having their effect through 7TMRs [210,211,212]. Among the multifaceted actions, GPCRs are involved in the regulation of vascularization and angiogenesis in the early stages of development as well as in tissue maturation and healing. In this scenario, the dissection of GPCR pharmacology may improve the current knowledge on the physio-pathological functions mediated by this heterogeneous class of receptors, and represents the ultimate goal for controlling the formation of aberrant blood vessel network, which is a distinguishing feature in the progression of inflammatory and ischemic diseases, as well as malignancies. Nevertheless, the pharmacological manipulation of GPCR-dependent pathways encounters some limitations due to the pleiotropic actions mediated by this broad class of membrane-embedded receptors. In this regard, the future challenge in the development of GPCR-targeted therapies is mainly represented by the identification of compounds with selective action and tailored action, to comply with GPCR pharmacogenomic. Molecular modeling, X-ray crystallography, in silico screening, protein bioengineering and new approaches aimed at dissecting the multi-dimensional features of GPCRs, represent crucial tools to narrow the pharmaceutical pipeline of GPCR-targeting agents in a clinical perspective.

**Figure 3:**
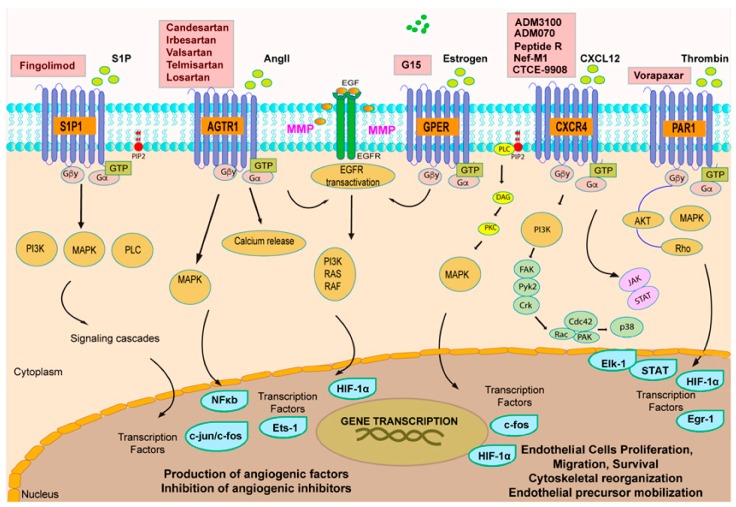
Schematic representation of the most relevant GPCRs, ligands, downstream pathways and candidate drugs involved in the regulation of the angiogenic process. S1P1, sphingosine 1 phosphate receptor 1; AGTR1, angiotensin II receptor type 1; GPER, G protein-estrogen receptor; EGFR, epidermal growth factor receptor; CXCR4, C-X-C chemokine receptor type 4; PAR1, protease activated receptor 1; S1P, sphingosine 1 phosphate; AngII, angiotensin II; EGFR, epidermal growth factor; CXCL12, C-X-C motif chemokine 12; GTP, guanosine triphosphate; PI3K, phosphoinositide 3-kinase; MAPK, mitogen-activated protein kinase; HIF-1α, hypoxia inducible factor-1 α; STAT, signal transducer and activator of transcription; FAK, focal adhesion kinase; EGR-1, early growth response protein 1; PIP2, phosphatidylinositol 4,5-bisphosphate; PLC, phospholipase C; DAG, diacylglycerol; MMP, matrix metalloproteinase; CDC42, cell division cycle 42; PAK, p21 activated kinase; RAC, ras-related c3 botulinum toxin substrate 1.

It should be mentioned that many GPCR-targeting compounds are currently proposed for the normalization of aberrant new blood vessel formation, in a large and heterogeneous range of pathological conditions (Figure 3). For instance, the pharmacological manipulation of CXCR4 pathway finds application in contrasting the detrimental effects of ischemic disease and myocardial infarction as well as in management of rheumatoid arthritis and liver fibrosis [213,214]. In this scenario, it is worth mentioning that several GPCR antagonists used to treat certain pathological states are now being re-purposed for their antiangiogenic properties. Indeed, the PAR1 antagonist vorapaxar, a well-known anti-platelet agent, has been proposed for the treatment of proliferative diabetic retinopathy (PDR) [47]. In addition, the Angiotensin II type 1 receptor antagonists (ARBs), used for the treatment of BP (blood pressure) and as renal- and cardio-protective agents, are currently regarded as novel pharmacological tools to normalize blood vessel network in cancer [52,53,138].

Representing the rate-limiting step in neoplastic growth and dissemination, tumor angiogenesis is finely regulated and achieved through multiple mechanisms that may enroll GPCR-mediated action as a pivotal molecular mechanism toward the acquisition of an aggressive phenotype [22]. In this context, combining the classical chemotherapeutic anti-cancer treatments with anti-angiogenic agents based on GPCR manipulation may represent an effective strategy for the eradication of neoplastic disease. Using GPCRs as drug candidates to halt the angiogenic process may pave the way for optimizing the current anti-angiogenic strategies, mainly based on disrupting VEGF/VEGFR2 axis. Indeed, the existing gap between the pre-clinical effects of anti-VEGF drugs respect to those observed in clinical models calls for further exploring how VEGF-targeting agents act in patients, in order to propose novel routes toward more effective and tailored anti-angiogenic therapy. To date, the clinical benefits derived from anti-VEGF therapy is limited by the activation of a plethora of compensatory biological responses [215]. For instance, the inhibition of VEGF signaling has been shown to induce the production of angiogenic chemokines and cytokines, including SDF-1 [216]. To overcome these limitations, a CXCR4-targeted lipid-based nanoparticle formulation has been shown to represent an effective approach for overcoming the evasion of anti-angiogenic therapy in hepatocellular carcinoma. In particular, the use of nanoparticles simultaneously delivering CXCR4 antagonist ADM3100 and siRNA sequences targeting the VEGF gene, has allowed to synergistically control tumor angiogenesis and prevent local and distant tumor growth [217].

Nevertheless, the generation transient hypoxia in response to anti-VEGF treatment, may trigger the mobilization of cancer stem cells (CSCs), thus limiting the clinical effectiveness of conventional angiogenic-impairing agents [218,219,220].

It is worth noting that the progression of certain metastatic diseases such as non-squamous non-small cell lung cancer (NSCLC) and ovarian cancer can be efficiently controlled using conventional anti-angiogenic agents, whereas such approach has consistently failed in impacting overall patient survival in metastatic breast, pancreatic, prostate cancer and melanoma [221]. It is plausible that relevant differences in tumor biology might be responsible for the different effects of classical anti-angiogenic strategy. Nevertheless, a further characterization of the multiple players involved in new blood vessel formation such as GPCRs may provide novel pharmacological targets to block the malignant evolution of neoplastic disease across different tumor types.

In the context of these observations, the manipulation of GPCR signaling may be strategically combined with classical anti-VEGF strategies to effectively halt the activation of angiogenic programs and block tumor progression. Such combination strategy holds promise for great therapeutic impact and deserves further consideration in order to improve the clinical management of cancer patients.

## Figures and Tables

**Table 1:** GPCRs involved in the regulation of vasculogenesis and angiogenesis in physiological conditions.

GPCR	Ligand	Target Cell/Tissue	Function	References
Frizzled 4, Frizzled 6, Frizzled 8	Wnt7a, 7b and Wnt2	ECs	BBB formation, CNS angiogenesis, hepatic angiogenesis	[24,25,26]
Frizzled 4, Frizzled 7	Wnt3A, Norrin	retinal ECs	retinal angiogenesis, BBB formation and maintenance	[27,28]
PAR1	Thrombin	mouse ESCs, human ECs, Platelets	vasculogenesis, angiogenesis	[29,30,31,32,33,34,35,36]
S1P1	S1P	ECs, VSMCs	vasculogenesis, angiogenesis	[37,38,39]
GPR126	Unknown	ECs	proliferation, migration, endothelial tube formation	[40]

BBB, brain blood barrier; EC, endothelial cell; ESCs, embryonic stem cells; VSMC, vascular smooth muscle cell; S1P, sphingosine-1-phosphate; GPR126, G protein receptor 126; PAR1, protease activated receptor 1.

**Table 2:** GPCRs involved in the regulation of angiogenesis in pathological conditions.

GPCR	Ligand	Target Cell/Tissue	Pathological Process	Function	References
S1P1	S1P	rat aortic smooth muscle cells, rat heart, renin containing mast cells	myocardial ischemia	protection against ischemic injury	[41,42,43]
S1P2	S1P	mouse retinal ECs	retinopathy	release of inflammatory mediators	[44]
PAR1	Thrombin	mouse aorta, ECs	acute myocardial infarction	angiogenesis	[45,46]
PAR1	Thrombin	human retinal microvascular ECs	proliferative diabetic retinopathy	cell proliferation	[47]
GPER	Unknown	rat heart	primary and secondary hypertension, myocardial ischemia	regulation of vascular tone and blood pressure	[48,49,50]

**Table 3:** GPCRs involved in the regulation of tumor angiogenesis.

GPCR	Ligand	Target Cell/Tissue	Function	References
LPAR1–LPAR3	LPA	ovarian cancer cells	activation of HIF/VEGF pathway	[51]
AGTR1	ANGII	TAMs, breast cancer cells	tumor angiogenesis, EMT	[52,53]
S1P1	S1P	TAMs	lymphangiogenesis	[54]
KSHV-GPCR	Orphan	ECs	tumor angiogenesis	[55,56]
CXCR4–7	CXCL12	cancer cells, CAFs	release of angiogenic factors, EPCs activation, angiogenesis	[57,58,59,60,61,62]
CXCR5	CCL5	chondrosarcoma cells, breast cancer cells	ECs differentiation, release of angiogenic factor	[63,64,65]
GPR124	Orphan	ECs	tumor angiogenesis	[66]
ELDT1	Orphan	tumor associated ECs	tumor angiogenesis	[67,68,69]
GPER	Orphan, 17β-Estradiol	breast cancer cells, CAFs	activation of HIF/VEGF pathway, tumor angiogenesis	[70,71,72]

TAM, tumor associate macrophages; CXCL12, C-X-C motif chemokine 12; CCL5, Chemokine C-C motif ligand 5; CXCR, C-X-C motif chemokine; ELDT1, EGF, latrophilin and seven transmembrane domain containing 1; KSHV-GPCR, Kaposi’s sarcoma-associated herpesvirus G protein-coupled receptor; AGTR1, angiotensin II receptor type 1; LPAR, lysophosphatidic acid receptor; HIF/VEGF, hypoxia-inducible factor/vascular endothelial growth factor; CAFs, cancer associated fibroblasts; EMT, epithelial mesenchymal transition; GPER, G protein-estrogen receptor.

## Abbreviations

GPCRs	G protein-Coupled Receptors
7TMRs	7 Transmembrane Receptors
GTP	Guanosine 5′-Triphosphate
VEGF	Vascular Endothelial Growth Factor
HIF-1	Hypoxia Inducible Factor 1
SDF-1	Stromal Derived Factor-1
S1P	Sphingosine 1-Phosphate
ELDT1	Epidermal Growth Factor, Latrophilin and Seven Transmembrane Domain-Containing Protein-1
GPER	G Protein Estrogen Receptor
PARs	Protease-Activated Receptors
LPARs	Lysophosphatidic Acid Receptors
AGTR	Angiotensin Receptor
VEGFR2	Vascular Endothelial Growth Factor Receptor 2
ROS	Reactive Oxygen Species
ECs	Endothelial Cells
EPCs	Endothelial Progenitor Cells
VSMCs	Vascular Smooth Muscle Cells
CAFs	Cancer Associated Fibroblasts
TAMs	Tumor Associated Macrophages
BBB	Blood–Brain Barrier
